# Qualitative assessment of knowledge, attitude and practice of oncologists about precision medicine in cancer patients- study from Lahore, Pakistan

**DOI:** 10.1371/journal.pone.0299010

**Published:** 2024-04-05

**Authors:** Rida Naaem, Furqan Khurshid Hashmi, Sulaman Yaqub, Dzul Azri Mohamed Noor

**Affiliations:** 1 Discipline of Clinical Pharmacy, School of Pharmaceutical Sciences, Universiti Sains Malaysia, Gelugor, Pulau Pinang, Malaysia; 2 University College of Pharmacy, University of the Punjab, Allama Iqbal Campus, Lahore, Pakistan; University of Hail, SAUDI ARABIA

## Abstract

**Background:**

Precision medicine (PM) is in great progressive stages in the West and allows healthcare practitioners (HCPs) to give treatment according to the patient’s genetic findings, physiological and environmental characteristics. PM is a relatively new treatment approach in Pakistan Therefore, it is important to investigate the level of awareness, attitude, and challenges faced by oncology physicians while practicing PM for various therapies, especially cancer treatment.

**Objectives:**

The present study aims to explore the level of awareness, attitude, and practice of PM in Pakistan along with the challenges faced by the oncologists for the treatment of cancer using the PM approach.

**Methods:**

Phenomenology-based qualitative approach was used. Face-to-face in-depth interviews were conducted using the purposive sampling approach among oncologists in Lahore, Pakistan. The data were analyzed using thematic content analysis to identify themes and sub-themes.

**Results:**

Out of 14 physicians interviewed 11 were aware of PM. They were keen on training to hone their skills and agreed on providing PM. Oncologists believed PM was expensive and given to affluent patients only. Other impeding factors include cost, lack of knowledge, and drug unavailability.

**Conclusions:**

Despite basic knowledge and will to practice, resource and cost constraints were marked as significant barriers. Additional training programs and inclusion into the curriculum may help to pave the way to PM implementation in the future. In addition, health authorities and policymakers need to ensure a cheaper PM treatment can be made available for all cancer patients.

## Introduction

Every individual responds differently to the same treatment even if they share the same disease history. Implementing precision medicine (PM) can prevent multiple treatment trials, thus preventing unnecessary treatment discomfort and pain [1–5]. PM is a molecular-level tailored treatment method that optimizes the therapeutic benefits by allowing healthcare practitioners (HCP) to select suitable treatment for the patient according to the patient’s genetic profile and the physiological and environmental disposition [4, 6]. In the Western world, it is much more in practice and, scientists and healthcare practitioners (HCPs) are making progress and discovering new knowledge every day, especially in chronic diseases such as cancer [7–13]. Unfortunately, this scenario is different in the lower- and middle-income countries (LMICs) due to the limitation of resources including financial constraints, knowledge, education, constricted practice opportunities as well as shortage of expertise [14–16].

PM is among those important treatment options for cancer patients that have yet not been thoroughly studied and implemented in Pakistan as many health practitioners in Pakistan still believe in conventional cancer treatment plans [17]. The role of healthcare practitioners (HCPs) especially physicians is very clear and important for PM implementation as reported by several studies around the world [18–22]. It is thus crucial to evaluate the awareness and practices of HCPs in Pakistan regarding PM as well as the resources required for its implementation especially when there is no published work present on the implementation of PM in Pakistan. This study will also provide background information for the policymakers on the barriers and challenges faced in implementing PM in Pakistan and also in deciding the best ways to implement PM completely in Pakistan.

## Materials and methods

### Study setting and design

A qualitative study was designed to explore the knowledge, attitude, and practice of healthcare practitioners regarding precision medicine in cancer patients for the first time in Pakistan. The study was conducted from December 2021 to February 2022.

In the current study, phenomenology was followed keeping in view the context and objectives of the research performed by monitoring the personified experiences of an individual to find a solution to the problem. A face-to-face in-depth, semi-structured interview approach was used for this purpose.

### Inclusion criteria

Pakistan Medical Council (PMC) registered oncologists working for a minimum of 8 hours daily in the oncology department of either government, or private or both types of hospitals in Lahore were included in the study.

All consented male and female candidates belonging to different age groups were given equal opportunity to participate and were treated equally.

### Ethics approval

The study was approved by the Punjab University Ethics Review Board Lahore, Pakistan (D/82/FIMS).

### Data collection tool and sampling

A semi-structured interview guide was developed based on the in-depth literature review [22–24] and current practices in the treatment of cancer in Pakistan. It was tested for face validity and content validity by two experts; one at the Punjab University College of Pharmacy (PUCP), Lahore, Pakistan who has PhD in pharmacy practice and holds vast experience in qualitative studies, and one MRCP oncologist at Hameed Latif Hospital Lahore who has extensive experience in clinical research. The validity of the interview guide was ensured by elaborated critical questioning and reasoning among the main researcher and research expert on each question included to ensure zero ambiguity in its content and meanings it presents and was pre-tested and verified for accuracy and consistency. Only a few grammatical changes were made after performing a pilot study in 2 physicians (S1 File). Later those participants were excluded from the study.

**Fig 1:**
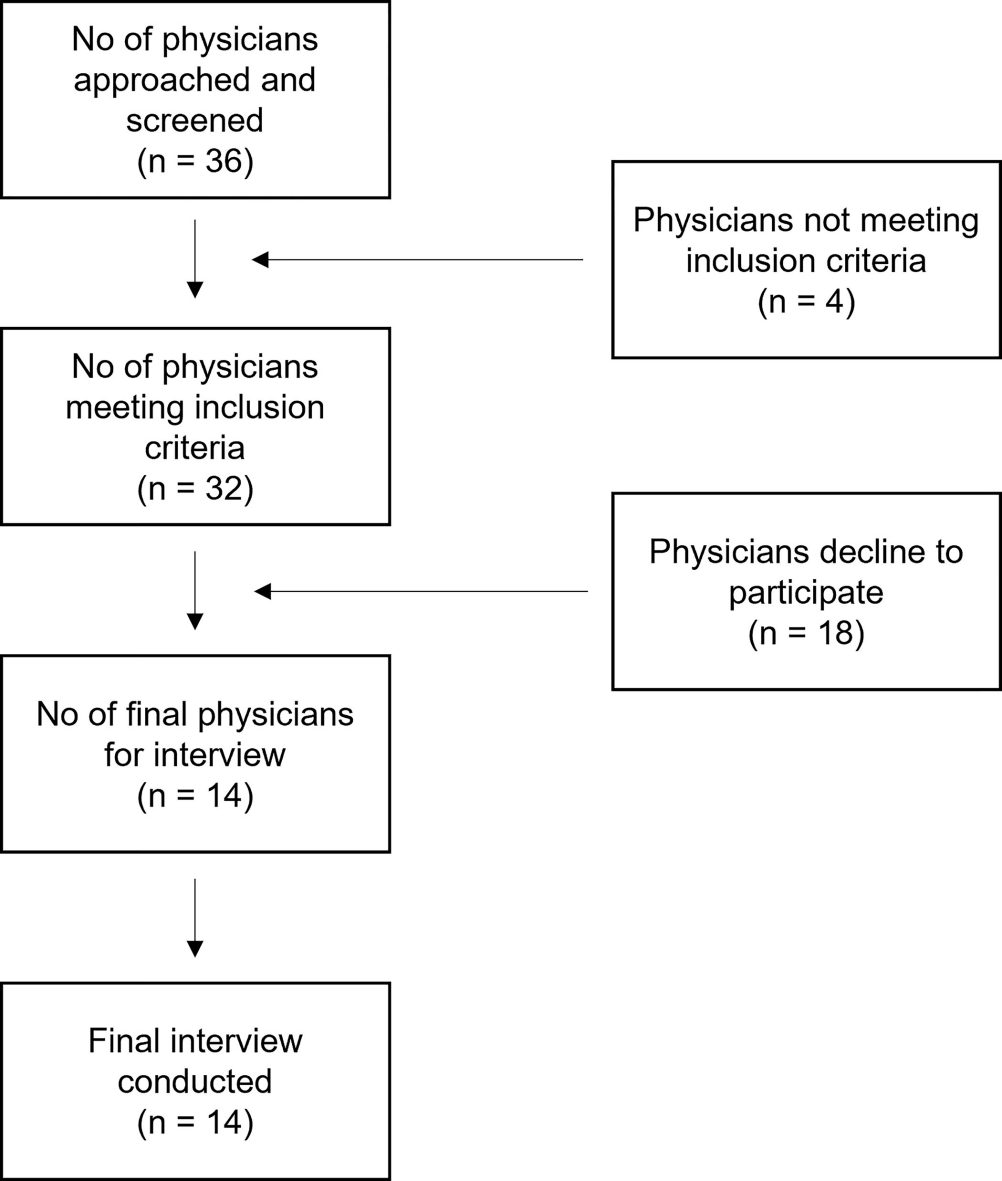
Flow chart of the study.

Participants were recruited using a purposive sampling technique for interviews. We approached 36 oncologists altogether (Fig 1) out of which, 14 were willing to participate. The rest of the targeted participants either did not fulfil any of the inclusion criteria or declined our request due to unstated reasons. Fourteen in-depth face-to-face interviews were conducted in the English language. All interviews were done at the working places of participants. Relevant probing was done to track down more information and the final sample size was determined after the saturation was achieved.

After giving an explanatory statement about the study objectives to the participants, written consent was taken from every individual. All interviews were audio recorded and each interview lasted about 20 to 25 minutes and were transcribed verbatim. Transcripts were not returned to participants for additional comments. All transcripts were individually further reviewed by two researchers for corrections and to ensure the credibility of the data collected.

### Analysis

Based on the method described by Braun and Clarke [25], inductive thematic analysis was performed by a thorough reading of every single interview manuscript and extracting all necessary information including emergent themes from them carefully. Data collected and verified was gone through manual thematic analysis upon finding codes to sort out main themes and sub-themes.

### Reporting

The reporting of the findings adheres to the consolidated criteria for reporting qualitative research (COREQ) checklist [26] (S1 Checklist).

## Results

### Demographics

An equal number of male and female participants took part in the interviews and most of them were from the age group 31 to 40 years. Around 64% had post-graduate degrees with more than 10 years of practicing experience in the relevant field. Participants included an equal number of oncologists from the government and private sectors. Almost half of them (43%) attend to around 50 patients daily with 8 hours per day working time. The demographic record of participants is presented in Table 1.

**Table 1:** Demographic characteristics of study participants (N = 14).

Demographic Character	Frequency (n)	Percentage
Age (years)		
· equal or less than 30	4	28.57
· 31–40	6	42.85
· 41–50	3	21.42
· 51–60	1	7.14
Gender		
· Male	7	50
· Female	7	50
Education		
· Graduate	5	35.71
· Post-graduate (FCPS)	9	64.28
Institution of graduation		
· Government	10	71.42
· Private	4	28.57
Years in practice		
· <5 years	4	28.57
· 5–10 years	4	28.57
· >10 years	6	42.85
Patients attended daily		
· <25	7	50
· 25–50	6	42.85
· >50	1	7.14
Job type (current)		
Government	5	35.71
Private	5	35.71
Both*	4	28.57

*Government and part-time private

### Thematic content analysis

Themes were derived manually after reviewing the literature. From the data obtained after being transcribed verbatim; sub-themes were derived from main themes.

The main themes knowledge, attitude and practice of healthcare practitioners for implementation of PM in Pakistan were further divided into a few sub-themes for in-depth investigation as summarized in Table 2.

**Table 2:** Thematic analysis of data.

Theme 1	Theme 2	Theme 3
Knowledge: of precision medicine (PM) in cancer treatment and other diseases	Attitude of healthcare practitioners (HCPs): Their willingness to adopt PM for cancer treatment	Practice of PM in Pakistan: Current practice of HCP and their hopes in the future
1.1 Basic knowledge of precision medicine and genomic testing • Awareness of basic concept of P.M • Other terms used than PM • Most treatable stage of cancer with PM	2.1 Determination to learn about the importance of PM use in cancer treatment: • Importance of knowing about its uses • Personal will to learn	3.1 Expected challenges and resources needed for implication • Challenges for implementation in Pakistan • Type of resources and support needed for implementation
1.2 Benefits of precision medicine in cancer • How is it helpful in cancer treatment • Benefit to HCS and patient	2.2 Willingness to implement (addressing patient’s general concerns) • Ability to discuss with the patient a treatment option • Address the patient’s religious and confidentiality concerns	3.2 Current practice of PM treatment and time required for complete implementation in Pakistan • Current working and preparedness of the oncology department to implement PM for cancer treatment • Number of years for complete implementation
1.3 Source of information • Formal education on PM • Other sources of information	2.3 Views on the cost of treatment at present and in the future	3.3 Implementation of PM as first-line treatment
		3.4 Need for betterment of physician’s role • Knowing and improving their role and its importance in HCS

#### Theme 1: Knowledge of PM in cancer treatment and other diseases

During face-to-face interviews, most of the oncology physicians were well-versed in precision medicine. Out of 14 physicians interviewed, only 3 did not have knowledge regarding this concept. And 3 out of 11 physicians demonstrated up-to-date and proficient knowledge. All three were working in government as well as part-time in a private hospital.

*Sub-theme 1*: *Basic knowledge of precision medicine and genomic testing*. Apart from very few physicians, all of them set forth a proper concept of PM which involves understanding the biology of tumor using genomic testing and then identifying a specific target drug for that tumor. They also mentioned terms used interchangeably with PM by the names of targeted therapy, gene therapy, and immunotherapy in almost all the setups. One of them stated:

“*Well*, *this term has been in circulation for quite some time now and by precision medicine term basically means in today’s era is the targeted therapies*. *They can be in the form of tablets*, *IV injections*, *in the form of immunotherapy*. *So*, *this is practically what it means*. *It usually targets a specific genetic mutation*, *or specific gene inside the patient’s body*, *inside the patient’s cell*.*”* PH#2

Two of them also highlighted its role with minimal side-effects:

“*When we know what we are targeting*, *we can prevent patient from the side effects of the therapy*” PH#11

A small number of physicians; 3 to be precise were unaware of this concept. Two of them were freshly graduated and new in the field. The other had been practicing in a government hospital for the last 19 years but did not even know that PM was partially being practiced in his hospital for quite some time.

When we asked which stage of cancer precision medicine could be most helpful, 10 of them agreed that its use and benefit vary with the type and stages of cancer.

“*In ninety-nine percent of cases in stage 4 solid tumors we can use precision medicine*. *But for hematological cancer*, *there is no need for any stage*.” PH#1

*Sub-theme 2*: *Benefits of precision medicine in cancer*. Young physicians with an average age of 30 and less than 5 practicing years were more influenced by adoption of individualized healthcare.

“*In precision medicine*, *we need to know targetable genes*. *We would have a more focused treatment approach according to target*. *We would be clearer where we are treating the patient and the patient would have less side effects in comparison to the chemotherapy”* [PH#4]

They also added that precision medicine also increases life expectancy.

“*It has produced a disease-free survival as well as progression-free survival advantage in various kinds of cancers*.” PH#9

Physicians between 30–40 years of age were hopeful that it would help cut down palliative care costs and decrease stay at hospitals.

“*If we know which specific drug has to be given*, *the cost can be reduced in terms that only that drug will be used*, *there will be better responses and less cost of palliative care*.” PH#11

*Sub-theme 3*: *Source of information*. All the young physicians mentioned that PM was not part of their MBBS curriculum. And among those with post-graduate education, most of them validated its inclusion in their FCPS training program.

“*It’s a part of our training*. *I did my FCPS in medical oncology and there is one subset of the topic of precision medicine in oncology*.” PH#1

Keeping aside formal education; physicians highlighted supportive sources of information. International conferences were named by almost all the participants. One source of information was “Molecular Tumor Boards”.

“*We do have a Molecular Tumor Board consisting of different presentations on different cases and agents being used in our every Saturday session*.*”* PH#8

#### Theme 2: Attitude of HCPs-their willingness to adopt PM for cancer treatment

Physicians’ willingness and motivation towards learning and implementing precision medicine were explored.

During interviews, a very optimistic and willing attitude was noticed. They were motivated to learn about new guidelines, genetic testing and the result interpretation, and other skills essential in PM practice.

*Sub-theme 1*: *Determination to learn about the importance of PM use in cancer treatment*. Physicians unraveled the importance of showing acceptable behavior towards learning and improving their knowledge and skills in PM practice, since the field of precision medicine is evolving every day.

“*In today’s world in medical oncology*. *If someone doesn’t know about precision medicine*, *I don’t think they are practicing properly*.” PH#2

*Sub-theme 2*: *Willingness to implement PM by addressing patient’s general concerns*. Few questions representing patient’s concerns were asked to evaluate the capacity of physicians for implementation of PM in the country.

All physicians showed optimistic gesture about counseling and convincing patients to choose PM based on the cost-effectiveness, better response rate than conventional therapy and survival benefits.

“*Yes I think patients are more convinced if you discuss about it in detail and tell them because its consequences are better than the conventional chemotherapy*.” PH#3

While responding to the queries of patients about involvement of genes and genetic testing; physicians stated a clear fact that?

“*Most of the tests are being done on biopsy samples as well as on the blood sample*. *So mostly none of these (confidentiality and religious sentiments) are breached*.” PH#3

*Sub-theme 3*: *Views on cost of treatment at present and in future*. All the participants established that PM is quite an expensive treatment option compared to other conventional therapies available.

“*So far*, *it’s very*, *very expensive*. *I don’t think (it is affordable)*. *It probably needs another 20*, *25 years*. *And it can be sort of cost cutting and cost saving*. *But right now*, *it’s pretty expensive for our healthcare or any healthcare right now*.” PH#2

However, some of them affirmed that:

“*It can only be cost saving if the drugs and tests are available at less price because if the cost of treatment is reduced*, *the burden of palliative and supportive care will also reduce*. *So*, *in that case the overall cost on the healthcare system will be reduced*.” PH#11

#### Theme 3: Practice of PM in Pakistan

Unfortunately, physicians were not very hopeful for complete implementation and availability of PM to every population in the country in coming times due to reasons explored below.

*Sub-theme 1*: *Expected challenges and resources needed for implication*. Cost, education and availability of drugs were identified to be the biggest challenges faced directly in Pakistan by physicians for PM implication.

“*Basically*, *funding is the big challenge*. *Otherwise*, *I think it should be available at every government as well as private sector*.” PH#4

“*Decreasing prices of medication for easy access and educating and training doctors about its uses*.*”* PH#10

It was brought into knowledge that the government is already supporting PM as treatment, but only for needy patient by using zakat funds and in collaboration with international pharmaceutical companies. But this fund is only enough to support a small number of patients.

“(Funding) *from Bait-ul-Maal zakat and by some NGOs can be utilized*.” PH#5

*Sub-theme 2*: *Current practice of PM treatment and time required for complete implementation in Pakistan*. Some of the respondents were ready to fully practice it; from sending patient for genetic investigations to deciding treatment strategy and prescribing targeted therapy while a few were reluctant due to resources constraints.

“*Currently due to cost issues we are not welcoming the people with warm hands about that Precision medicine*.*”* And *“I think I can answer that question only from the side of oncology people*. *Yes*, *oncology people are ready*, *but I don’t know about other physicians or other pharmacists*.*”* PH#7

However, the status of PM practice is in place to some extent in some of the specialized cancer hospitals in the private sector.

“*Exclusive cancer setup they are well organized*.” PH#12

Different number of years were predicted by the physicians for complete implementation of PM in Pakistan but on average 10 years were forecasted except one who mentioned that he and his hospital is already practicing it. A few recited 5–10 and others quoted 8–10 years. Only two of them mentioned less than 5 years. And one highly experienced personnel stated 25 years for complete implementation in the country.

“*I hope in the next 10 years*, *we will be at some point of providing this facility to our all patients*.*”* PH# 6

*Sub-theme 3*: *Implementation of PM as first-line treatment*. Nearly all physicians except one showed a positive response towards the use of precision medicine as first-line treatment.

“*In Pakistan most stage 4 cancers like lung cancer which is positive for EGFR can be treated with osimertinib (as first line treatment drug) and if it is positive for ALK they can be treated with ALK inhibitors”* PH#5

Two of them were aware about its use in first line treatment. But they were more dependent upon its research and more clinical trials results.

“*Right now*, *no*, *but in the future*, *if results are better in stage 4 or stage three tumors*, *then it can be a part of treatment first-line management*.” PH#7.

*Sub-theme 4*: *Need for betterment of physician’s role for implementation of PM in Pakistan*. Most of the physicians spelled out fundamentals to bring better policies and better treatment services to their system and people with their skills and knowledge.

“*All the health care workers including general physicians in tertiary cares must be aware at least*. *And when and where and why this must be known to everyone*.” PH#9

“*I think they should be guided*. *I think it should be part of the basic curriculum for majority of the fellowship*, *examinations”* PH#5

One of them highlighted an important necessity that must be addressed by physicians for the betterment of their patients’ i.e., patient counseling.

“*I would say that a health care practitioner should not be only dealing with the patient or the disease itself*. *It should be a general or whole idea about that patient*. *How psychologically or how generally*, *that patient is dealing with the disease and not only the disease state*.*”* PH#4

## Discussion

Better knowledge and improved attitude of healthcare practitioners lead to better implementation of precision medicine in any country’s healthcare system [27, 28]. The same can be observed with the healthcare system (HCS) of Pakistan and healthcare practitioners (HCPs) specifically working currently in oncology. This study, allowed the oncologists of Lahore, Pakistan to express their views and practices on PM in cancer treatment. They were given chance to share the barriers and challenges faced by them while practicing PM in Pakistan. This study comprehends all the influential elements, including knowledge, attitude, determination, practice, restrictions encountered, and resources &policies needed to enforce and advance in cancer treatment using precision medicine.

Present study revealed better knowledge of physicians on PM concept even if partially practiced in our country as compared to studies performed around the world [22]. Several studies have proved that HCPs comprehension and frame of mind has a lot to do with precision medicine establishment and each one of them evaluated the knowledge and attitude of physicians; the most powerful tool to access PM in the country’s HCS [24, 29, 30]. In this study physicians aged more than 30 years and with an experience of more than 10 years showed better knowledge on the use of precision medicine in oncology than the younger ones in the field with less experience. This was contrary to the studies conducted in Canada where young and recently graduated doctors had better knowledge on progress in PM and better trained to employ it in cancer treatment as compared to senior ones [31]. It was also brought into knowledge that precision medicine was known by other alternative terms in Pakistan like targeted therapy, immunotherapy, and personalized medicine. Physicians were more comfortable and expressive when any of these alternative terms were used. However, similarities and differences among these terms were not discussed during this research. All these terms have been coined to use alternatively with PM for quite some times and are still under discussion to use this way [32].

Fresh physicians showed poor knowledge and a lack of interest comparatively and this could be attributed by the absence of PM topics in their undergraduate curriculum. Thus it is suggested to include precision medicine education as part of the MBBS curriculum and their training in a similar manner as suggested in earlier researches [8, 33]. A study performed in Korea in 2019 showed similar result that despite the promising attitude of HCPs toward PM implementation, they had concerns about HCPs education and attainment of knowledge [23]. A study performed by Nagy in Cairo, Egypt also identified several challenges faced in paving the road for PM implementation including limited education resources as one of the biggest challenges [18]. Therefore, credible and accessible sources of information should be provided to physicians to upgrade their knowledge on a regular basis.

The current study also concluded that physicians are eager to implement precision medicine in the country for cancer treatment. They showed an optimistic attitude like other physicians of the world [34]. They were willing to learn more about it and practice it in a better way in their setup and at a bigger scale if properly guided and facilitated since all of them believe in its better outcomes than conventional therapy just like their fellow oncologists around the world [18, 20, 23]. Most of them are willing to be involved in PM research to gain more knowledge and skills regarding it. They also showed an optimistic response toward revolution to be brought about by PM and pharmacogenomics testing [35].

Our project strongly suggested the need for physicians to learn about PM and excel in this field with their competency that lies in the fact that physicians should be able to convince patients and guide them about better outcomes of PM as compared to conventional therapy. Similarly, a survey in Qatar done in 2016 highlighted the importance of physicians being constantly updated on the diagnostics, treatment guidelines, strategies of PM, and dealing with patients [24].

No conflict of interest was found on patient data confidentiality or hurting religious sentiments by any of the HCPs during research. All of them agreed that PM does not intervene with patient’s personal information or religious principles. Since sampling is done just the same as for other diagnostic tests and results or data obtained fall under the hospital’s responsibility. It is not shared with anyone. Our study results somehow contradict the study done on this aspect in the past. Like in a study performed on the Alaska Native and American Indian community (ANAI), the positive shift of attitude and experience was reported by healthcare providers in the overall health system due to the introduction of PM but privacy and transparency of using genetic information issues were considered as a serious threat for future implementation of PM [36]. [27] Rahma also published doubt of HCPs on the involvement of religious concerns in PM implication.

All of the physicians interviewed, declared financial burden and cost of treatment to be the biggest challenge they face just like the findings published in South East Asia [37]. The financial burden was segregated into different forms including the healthcare budget of the country, testing facilities, and manufacturing, providing targets locally and at cheaper prices, treatment costs and palliative care costs. To overcome this, financial support should be provided by the government as covered by public insurance in other countries [38], NGOs, the pharmaceutical sector [33], and financially sound people of society.

During inquiry, we explored a thriving fact that Pakistan Bait-ul-Maal and zakat funds (Zakat is the fifth pillar of Islam based on the fiscal act. It is the due-share of poor people of Muslim society in the wealth of affluent people of society to get basic living facilities including food, shelter, health and education [39] from government is already financially aiding cancer patients utilizing precision medicine. Bait-ul-Maal is a social safety net in Pakistan financed by the government of Pakistan as well as the general public that provides financial assistance and help to needy and deprived groups of society through different programs [40]. They have an “Individual Financial Assistance” Program (IFA) and Chronic Myelogenous Leukemia CML project with a renowned pharmaceutical company for this purpose, but this program is currently running at a small scale due to limited resources available. They provide patients with medication as well as help in providing testing facilities. Thus, a similar approach or program can be further explored to provide patients a better access to PM treatment.

Most of the physicians anticipated that it may take around 10 years for complete implementation. Likewise, a study was conducted on pharmacogenomics testing in Pakistan during 2019–2020 in which HCPs foresaw the introduction of pharmacogenomics testing in the next 10 years in Pakistan [41].

It was encouraging to know about the current practice of PM in Pakistan with the use of first-line therapy in different stage 4 metastatic cancers and as a diagnostic tool. A few of the examples quoted include Osimertinib for EGFR mutated NSCLC (non-small-cell lung cancer) and ALK (anaplastic lymphoma kinase) inhibitors for ALK-positive NSCLC. Studies have already proven their better outcomes than conventional chemotherapy and use as preference as first-line treatment [42–44]. These factors may pave the way towards early implementation in the country. Therefore, with more published research data, a wider implementation of PM can be predicted.

Commenting on their role; some of the physicians shared their views on providing education & training to their other physician colleagues who must be vigilant enough to know each and everything about PM when needed. A similar suggestion was provided by Wang in his study [45] Not just this they should play an active role in patients. This role shall help patients understand their disease better and aid them in deciding on their treatment.

### Limitations

This study was conducted within a specific period and is restricted to oncologists working in hospitals of Lahore, Pakistan. Hence, the results obtained are not generalized and cannot be undoubtedly anticipated towards whole country. Furthermore, the results might represent the healthcare practitioner’s point of view at a particular point of time but trend over time was not observed. Besides, no historical data with this perspective in the country was present at that time. Furthermore, other healthcare professionals such as pharmacists and nurses are not included in the current study. Thus, this study may not represent the view of the whole healthcare professional community.

## Conclusion

Study findings suggest that the PM topic should be included as an integral part of the MBBS curriculum to bring awareness to upcoming doctors at an early level. Molecular tumor board should be practiced at every cancer hospital to increase the exposure of HCPs towards PM and its practice. But above all, resources and funding must be provided at government and private levels. Pharmaceutical companies can play an important role in this aspect by locally manufacturing and selling “targets” at cheaper price and by collaborating with the government and relevant authorities to bring genetic testing to Pakistan and eventually allow its full implementation in the country.

## Supporting information

S1 Checklist COREQ checklist. (DOCX)

S1 File Interview guide. (DOCX)
