# Supplementary material for: Qualitative assessment of knowledge, attitude and practice of oncologists about precision medicine in cancer patients- study from Lahore, Pakistan
Source: PLoS One. 2024 Apr 5;19(4):e0299010. doi: 10.1371/journal.pone.0299010 (PMC10997134; doi:10.1371/journal.pone.0299010)
Supplement: S1 Checklist — (DOCX) [file pone.0299010.s001.docx]

**SUPPLEMENTARY MATERIAL**

**SUPPLEMENTARY 2: COREQ Checklist**

| **No.** | **Item** | **Description** | **Section #** |
| --- | --- | --- | --- |
| **Domain 1: Research team and reflexivity** | | |  |
| Personal Characteristics | | |  |
| *1.* | Interviewer/facilitator | Which author/s conducted the interview or focus group? | Rida Naeem |
| *2.* | Credentials | What were the researcher's credentials? *E.g. PhD, MD* | Student MS Clinical Pharmacy |
| *3.* | Occupation | What was their occupation at the time of the study? | Researcher |
| *4.* | Gender | Was the researcher male or female? | Female |
| *5.* | Experience and training | What experience or training did the researcher have? | Already conducted quantitative research using a survey questionnaire |
| Relationship with participants | | |  |
| *6.* | Relationship established | Was a relationship established before the study commencement? | Participants were informed about the purpose of the study |
| *7.* | Participant knowledge of the interviewer | What did the participants know about the researcher? *E.g. Personal goals, reasons for doing the research* | Participants were informed about the basic theme of conducting the research and consent was also taken from them |
| *8.* | Interviewer characteristics | What characteristics were reported about the interviewer/facilitator? *E.g. Bias, assumptions, reasons, and interests in the research topic* | - |
| **Domain 2: Study design** | | |  |
| Theoretical framework | | |  |
| *9.* | Methodological orientation and theory | What methodological orientation was stated to underpin the study? *E.g. grounded theory, discourse analysis, ethnography, phenomenology, content analysis* | Phenomenology |
| Participant selection | | |  |
| *10.* | Sampling | How were participants selected? *E.g. purposive, convenience, consecutive, snowball* | Purposive sampling |
| *11.* | Method of approach | How were participants approached? *E.g. face-to-face, telephone, mail, email* | Face-to-face |
| *12.* | Sample size | How many participants were in the study? | 14 |
| *13.* | Non-participation | How many people refused to participate or dropped out? What were the reasons for this? | 22 (did not fulfil any of the inclusion criteria or declined our request due to unstated reasons) |
| Setting | | |  |
| *14.* | The setting of data collection | Where was the data collected? *E.g. home, clinic, workplace* | At their workplaces |
| *15.* | Presence of nonparticipants | Was anyone else present besides the participants and researchers? | No |
| *16.* | Description of sample | What are the important characteristics of the sample? *E.g. demographic data, date* | Participants must be oncologists only |
| Data collection | | | |
| *17.* | Interview guide | Were questions, prompts, and guides provided by the authors? Was it pilot-tested? | Yes |
| *18.* | Repeat interviews | Were repeat interviews carried out? If yes, how many? | No |
| *19.* | Audio/visual recording | Did the research use audio or visual recording to collect the data? | Audio recording |
| *20.* | Field notes | Were field notes made during and/or after the interview or focus group? | Yes |
| *21.* | Duration | What was the duration of the interviews or focus groups? | 20-25 minutes |
| *22.* | Data saturation | Was data saturation discussed? | Yes |
| *23.* | Transcripts returned | Were transcripts returned to participants for comment and/or correction? | No |
| **Domain 3: analysis and findings** | | | |
| Data analysis | | | |
| *24.* | Number of data coders | How many data coders coded the data? | 1 |
| *25.* | Description of the coding tree | Did the authors provide a description of the coding tree? | Yes |
| *26.* | Derivation of themes | Were themes identified in advance or derived from the data? | Derived |
| *27.* | Software | What software, if applicable, was used to manage the data? | Manual thematic analysis performed |
| *28.* | Participant checking | Did participants provide feedback on the findings? | Not needed |
| Reporting | | | |
| *29.* | Quotations presented | Were participant quotations presented to illustrate the themes/findings? Was each quotation identified? *E.g. Participant number* | Every participant was coded |
| *30.* | Data and findings consistent | Was there consistency between the data presented and the findings? | Yes |
| *31.* | Clarity of major themes | Were major themes clearly presented in the findings? | Yes |
| *32.* | Clarity of minor themes | Is there a description of diverse cases or a discussion of minor themes? | Sub-themes described |
