# Supplementary material for: Qualitative assessment of knowledge, attitude and practice of oncologists about precision medicine in cancer patients- study from Lahore, Pakistan
Source: PLoS One. 2024 Apr 5;19(4):e0299010. doi: 10.1371/journal.pone.0299010 (PMC10997134; doi:10.1371/journal.pone.0299010)
Supplement: S1 File — (DOCX) [file pone.0299010.s002.docx]

**SUPPLEMENTARY MATERIAL**

**SUPPLEMENTARY 1: INTERVIEW GUIDE**

**MIXED METHODOLOGICAL ASSESSMENT OF KNOWLEDGE, ATTITUDE AND PRACTICE OF HEALTHCARE PRACTITIONERS ABOUT PRECISION MEDICINE IN CANCER PATIENTS, LAHORE PAKISTAN**

**PARTICIPANTS**: Physicians

**Knowledge:**

1. Are you aware of precision medicine in practice?
2. Do you have any idea it’s been used in Pakistan?
3. Does your hospital utilize precision medicine?
4. Are you aware of the applications of precision medicine in daily routine and what is its significance?
5. Do you think PM would be helpful in cancer treatment and how?
6. Did you ever get any formal education and training on precision medicine? (Probe in: Sources of information)
7. Can precision medicine really be viewed as end-point of a novel stratification of patients, as implied, or is it rather a greater whole?
8. Do you think it is possible in some circumstances for a person to have their tumor tested to help their doctor decide which treatment to give?
9. Can you specify for which stage cancer patient precision medicine can be used?

**Attitude and barriers faced:**

1. Do you think it is important to know about its use?(What approach is needed by healthcare practitioner to implement it?
2. Are you willing to know and learn more about it?
3. In your opinion is treatment decision a shared decision-making process between doctor, and/or multidisciplinary team and patient?

(Probe in: What do you think is the role of physician/pharmacist in decision making on precision medicine?)

1. Do you think you can discuss it with your patient as a treatment option and convince him about it?
2. Do you think patient’s confidentiality is breached? (Probe in: Do you think it has something to do with religion? Is it going to hurt anyone religiously?)
3. Can it be influenced by physical examination?
4. Do you think it can cause any sort of mutation and worsen the disease state?
5. Do you personally believe PM is beneficial to healthcare system and patients?
6. What are your views on its education in community? (Probe in: How important it is to join patient support group to implement PM)
7. We know cost is a big issue in oncology for patients and payers. Do you think that this new technology can be cost saving?

**Practice and Future Concern**

1. What kind of support and resources you look for in terms of funding for its implementation in your country’s healthcare system?
2. Can it be used as first line treatment in cancer treatment?
3. How well oncology department services are prepared to deliver humane and warm response to precision medicine in Pakistan? (Probe in: How can we improve it?)
4. What will change as we begin to catch disease earlier using precision medicine?
5. And how long will it take to apply precision medicine in Pakistan?
6. Do you think for any patient with precision medicine, day to day management of cancer is needed?
7. **Enablers of precision medicine in Pakistan:**
8. What type of resources would you want to help you make decisions about including precision medicine in your healthcare?
9. Enablers of precision medicine in Pakistan:
   1. What are the big challenges for its implementation in Pakistan?
   2. What are the potential draw backs to its approach?
   3. Do you think it will have global healthcare implications as well as enhancing personalized well-being?

**Conclusion**

Any additional comments about role of healthcare practitioner in practicing precision medicine in Pakistan?
